# A recombinant *Bifidobacterium bifidum* BGN4 strain expressing the streptococcal superoxide dismutase gene ameliorates inflammatory bowel disease

**DOI:** 10.1186/s12934-022-01840-2

**Published:** 2022-06-07

**Authors:** Sini Kang, Zhaoyan Lin, Yang Xu, Minju Park, Geun Eog Ji, Tony V. Johnston, Seockmo Ku, Myeong Soo Park

**Affiliations:** 1 Key Laboratory of Fermentation Engineering (Ministry of Education), National “111” Center for Cellular Regulation and Molecular Pharmaceutics, Cooperative Innovation Center of Industrial Fermentation (Ministry of Education & Hubei Province), Hubei Key Laboratory of Industrial Microbiology, Hubei University of Technology, Wuhan, 430068 China; 2 Department of Food and Nutrition, Research Institute of Human Ecology, Seoul National University, Seoul, 08826 South Korea; 3 Research Center, BIFIDO Co., Ltd, Hongcheon, 25117 South Korea; 4 Fermentation Science Program, School of Agriculture, College of Basic and Applied Sciences, Middle Tennessee State University, Murfreesboro, TN 37132 USA

**Keywords:** Inflammatory bowel disease, *Bifidobacterium bifidum*, Catalase, Superoxide peroxidase, Human interleukin-10

## Abstract

**Background:**

Inflammatory bowel disease (IBD) is a gastrointestinal disease characterized by diarrhea, rectal bleeding, abdominal pain, and weight loss. Recombinant probiotics producing specific proteins with IBD therapeutic potential are currently considered novel drug substitutes. In this study, a *Bifidobacterium bifidum* BGN4-SK strain was designed to produce the antioxidant enzymes streptococcal superoxide dismutase (SOD) and lactobacillus catalase (CAT), and a *B. bifidum* BGN4-pBESIL10 strain was proposed to generate an anti-inflammatory cytokine, human interleukin (IL)-10. In vitro and in vivo efficacy of these genetically modified *Bifidobacterium* strains were evaluated for colitis amelioration.

**Results:**

In a lipopolysaccharide (LPS)-stimulated HT-29 cell model, tumor necrosis factor (TNF)-α and IL-8 production was significantly suppressed in the *B. bifidum* BGN4-SK treatment, followed by *B. bifidum* BGN4-pBESIL10 treatment, when compared to the LPS-treated control. Synergistic effects on TNF-α suppression were also observed. In a dextran sodium sulphate (DSS)-induced colitis mouse model, *B. bifidum* BGN4-SK treatment significantly enhanced levels of antioxidant enzymes SOD, glutathione peroxidase (GSH-Px) and CAT, compared to the DSS-only group. *B. bifidum* BGN4-SK significantly ameliorated the symptoms of DSS-induced colitis, increased the expression of tight junction genes (claudin and ZO-1), and decreased pro-inflammatory cytokines IL-6, IL-1β and TNF-α.

**Conclusions:**

These findings suggest that *B. bifidum* BGN4-SK ameliorated DSS-induced colitis by generating antioxidant enzymes, maintaining the epithelial barrier, and decreasing the production of pro-inflammatory cytokines. Although *B. bifidum* BGN4-pBESIL10 exerted anti-inflammatory effects in vitro, the enhancement of IL-10 production and alleviation of colitis were very limited.

**Supplementary Information:**

The online version contains supplementary material available at 10.1186/s12934-022-01840-2.

## Introduction

Inflammatory bowel disease (IBD) is a chronic inflammatory disease with symptoms that include diarrhea, rectal bleeding, abdominal pain over an extended period of time. Though IBD is not a fatal disease, it can noticeably decrease quality of life and enhance the risk of colorectal cancer [1]. Probiotics are live microorganisms that, when administered in adequate amounts, confer health benefit(s) to the host [2]. Though many clinical trials of probiotic therapies against IBD have been conducted, the results have been inconsistent [3–6]. To further augment the beneficial effects of probiotics, genetic modification can be used to construct recombinant strains that secrete specific anti-inflammatory proteins and offer additional beneficial effects [7]. The most frequently utilized engineered probiotics include *Lactococcus*, *Lactobacillus sensu strictu*, *Bifidobacterium*, and *Escherichia coli* Nissle 1917. Their colonization capabilities make them potential candidates as carrier organisms for synthesized therapeutic molecules *in situ*, and their use can reduce side effects compared to the injection of medication or refined drugs [8]. For instance, IBD requires lifelong medication. It is highly desirable to develop inexpensive, easily administered therapeutics with minimal side effects [9]. Thus, the use of genetically modified probiotics with anti-inflammatory potential can be an effective strategy for IBD treatment [7].

IBD is noted for reactive oxygen species (ROS)-induced oxidative stress generated by neutrophils and macrophages in the inflamed epithelial tissues, with reduced antioxidant capacity in the plasma [10]. Previous studies have indicated that more oxygen free radicals are generated in the colons of IBD patients than those of healthy subjects, and the imbalance between prooxidant and antioxidant compounds leads to the development of IBD [11, 12]. Tissues respond to mild oxidative stress by producing more antioxidants. When oxidative stress becomes severe and persistent, antioxidant reserves in the tissues are exhausted and their capability for antioxidant generation is diminished, resulting in lower antioxidant levels and tissue injury [13]. Usually, superoxide dismutase (SOD) and catalase (CAT) are recruited as antioxidative strategies for reducing IBD inflammatory damage. Specifically, SOD converts the highly reactive superoxide anion O^2−^ to the less reactive species H_2_O_2_, then CAT catabolizes the hydrogen peroxide into O_2_ and H_2_O [14]. Several recombinant SOD/CAT-expressing lactic acid bacteria have been developed with the capability to reduce inflammation, as demonstrated in different murine models of chemically induced colitis with diminished ROS levels in the gut [15–18].

Interleukin (IL)-10 is a critical anti-inflammatory cytokine involved in the maintenance of intestinal immune responses, protecting hosts from an excessive response to inflammation [19]. Oral administration of human IL-10 protein is not feasible because it is unstable and destroyed transiting the human gastrointestinal tract [20]. Genetically modified probiotic bacteria have been developed to surge intestinal IL-10 levels via local protein delivery or DNA delivery systems that trigger DNA expression by intestinal cells to produce IL-10 directly at the site of inflammation [7, 21]. The first study related to genetically modified probiotics expressing IL-10 was conducted in 2000. Recombinant *Lactococcus lactis* expressing murine IL-10 protected IL-10^−/−^ mice from colitis and significantly reduced inflammation in mouse colitis induced by dextran sodium sulphate (DSS) [22].

Research related to genetically modified bacteria has increasingly focused on the direct oral administration of these recombinant bacteria to humans mainly for *in situ* delivery of proteins of therapeutic interest (i.e., antioxidants, cytokines, and protease inhibitors) [23, 24]. In this study, *Bifidobacterium bifidum* BGN4 was genetically modified as *B. bifidum* BGN4-SK to produce *streptococcal* SOD and *lactobacillus* CAT, and *B. bifidum* BGN4-pBESIL10 to produce human IL-10. Although *Bifidobacterium* sp. offer host health benefits, they are limited as genetically modified bacteria by their strict anaerobic metabolism, multilayered and complex cell walls, and restriction–modification systems. Thus, compared to genetically modified lactic acid bacteria, recombinant *Bifidobacteria* spp. have only recently been developed [25]. Based on our previous studies, recombinant *B. bifidum* BGN4-SK was constructed by introducing the SOD (*StSodA*) and catalase (*LpKatL*) genes into *B. bifidum* BGN4 [26]. *Bifidobacterium bifidum* BGN4-pBESIL10 was constructed by cloning the human IL-10 gene into the *E. coli-Bifidobacterium* shuttle vector pBES2 [27]. The aim of this study was to evaluate the immunomodulatory effects of *B. bifidum* BGN4-SK, *B. bifidum* BGN4-pBESIL10, and their combination in both lipopolysaccharide (LPS)-stimulated HT-29 cell and DSS-induced mouse colitis models, and to compare their effects to that of a control bacterial strain.

## Materials and methods

### Preparation of bacterial strains

*Bifidobacterium bifidum* BGN4, *B. bifidum* BGN4-SK and *B. bifidum* BGN4-pBESIL10 were cultured in MRS medium containing 0.05% l-cysteine·HCl, 30 µM hematin and 500 µM MnSO_4_ at 37 ºC for 24 h under anaerobic conditions [27].

### Anti-inflammatory effects of *B. bifidum* BGN4-SK and* B. bifidum *BGN4-pBESIL10 in a cell line model

#### Cell line preparation

The HT-29 (KCLB 30,038) cell line was purchased from the Korea Cell Line Bank (Seoul, Korea). The cells were cultured in Dulbecco’s Modified Eagle’s Medium (DMEM, Gibco, NY, USA) supplemented with 10% (v/v) heat-inactivated fetal bovine serum (Gibco) and 1% penicillin/streptomycin (Sigma Aldrich, USA) at 37 ºC in an atmosphere of 5% CO_2_. Thereafter, HT-29 cells were seeded into 24-well plates at a density of 1 × 10^6^ cells per well and cultured for 24 h at 37 ºC.

#### Determination of cytokines

*Bifidobacterium bifidum* BGN4-SK and *B. bifidum* BGN4-pBESIL10 were adjusted to 10^8^ CFU/mL and the HT-29 cells were treated with 100 ng/mL LPS from *Escherichia coli* 055: B5 (Sigma Aldrich, USA), and 100 µL neutralized cell-free supernatants (CFSs) of *B. bifidum* BGN4-SK, *B. bifidum* BGN4-pBESIL10 or a mixture of 50% (v/v) *B. bifidum* BGN4-SK and 50% (v/v) *B. bifidum* BGN4-pBESIL10, followed by incubation for 10 h at 37 ºC. Then, cell culture supernatants were collected to determine the levels of tumor necrosis factor (TNF)-α and IL-8 using enzyme-linked immunosorbent assay kits (BD Biosciences, CA, USA), according to the manufacturer’s instructions.

### Anti-inflammatory effects of *B. bifidum* BGN4-SK and *B. bifidum* BGN4-pBESIL10 in a DSS-induced colitis model

#### Animals and treatments

Facilities and protocols employed in this study were approved by the Institutional Animal Care and Use Committee of Seoul National University (Approval Number SNU-200529-1). Forty-eight 8-week-old female C57BL/6 mice were obtained from Central Lab Animal, Inc. (Sungnam, Korea). All mice had free access to American Institute of Nutrition-93G diet (Doo Yeol Biotech Co., Lid., Korea). After acclimatization to the environment for 1 week, the mice were randomly assigned to six groups (n = 8 per group): untreated control (Control), DSS-treated group (DSS), 10^10^ CFU lyophilized *B. bifidum* BGN4 treatment (BGN4), 10^10^ CFU lyophilized *B. bifidum* BGN4-SK treatment (SK), 10^10^ CFU lyophilized *B. bifidum* BGN4-pBESIL10 treatment (IL-10) and a mixture of 5 × 10^9^ CFU *B. bifidum* BGN4-SK and 5 × 10^9^ CFU *B. bifidum* BGN4-pBESIL10 (SK + IL-10). The probiotic cultures were lyophilized for 48 h in a Virtis Freezemobile 12EL (SP Scientific, NY). Each lyophilized bifidobacterial stock was tested for bacterial viability and enumeration before oral administration, and the bifidobacterial powder was resuspended daily in the sterilized water for administration to each mouse.

**Fig. 1:**
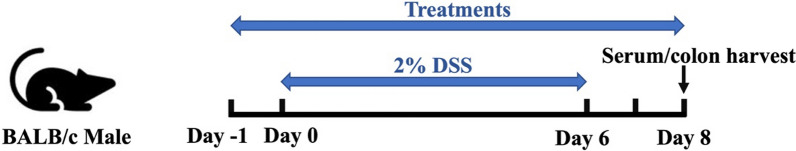
Schematic overview of the murine model of DSS-induced colitis

The DSS-induced colitis groups received 2% (w/v) DSS (36–50 kDa) (MP Biomedical, CA, USA) in drinking water *ad libitum* for 6 days and then drank sterilized water for two days before sacrifice. Treatment groups were orally administered lyophilized *B. bifidum* strains every day, by gavage, starting one day prior to DSS induction, while the control and the DSS groups received PBS solution at the same volume, as shown in Fig. 1. All mice were euthanized by CO_2_ asphyxiation.

#### Antioxidant enzyme activities

Blood samples were collected into 1.5 mL heparinized tubes by cardiac puncture. The blood was centrifuged at 1000×*g* for 10 min at 4 ºC to harvest serum samples. To determine serum antioxidant enzyme activities, SOD assay, catalase assay and GSH-Px assay kits were utilized according to the manufacturer’s instructions.

#### Histological analysis

Colon tissues were collected immediately after animal sacrifice. The colonic tissues of three mice per group were prepared as “Swiss rolls” for histological analysis and fixed in 10% buffered formalin, dehydrated in ethanol, and then embedded in paraffin. Then, 5 mm sections were prepared and stained with hematoxylin and eosin and the samples were rated on a scale of 0–4 for the extent of crypt damage, epithelial injury, and inflammatory infiltration (0 = none; 1 = mild; 2 = moderate; 3 = severe; and 4 = very severe).

#### Tissue myeloperoxidase (MPO) activity

Proximal colonic tissues were homogenized and evaluated for myeloperoxidase (MPO) activity by Myeloperoxidase assay kit (Hycult Biotech, Wayne, PA, USA) according to the manufacturer’s instruction.

#### Real-time polymerase chain reaction (PCR) of the colonic tissues

The collected colonic tissues (30 mg) were homogenized in 600 µL of RNAlater with TissueLyser II (Qiagen, CA, USA) for 2 min at 50 Hz. Total RNA was isolated from the homogenized tissues using RNeasy Mini kits (Qiagen, CA, USA) following the protocol’s guidance. This extraction system includes the DNase treatment to eliminate genomic DNA. The purity and quantity of obtained RNA were verified using a NanoDrop™ ND-1000 Spectrophotometer (Thermo Fisher Scientific Inc., MA, USA). Extracted RNA was reverse transcribed into cDNA using a GoScript™ Reverse Transcription kit (Promega, WI, USA). Quantitative real-time PCR was conducted with a StepOne™ Real-time PCR System (Applied Biosystems, CA, USA) using SYBR Green PCR Master Mix (Applied Biosystems). qRT-PCR was performed as following: 2 min at 95 ºC for initiation, 15 s at 95 ºC for denaturation and 60 s at 60 ºC for annealing up to 38 cycles. All qRT-PCR reactions were completed in triplicate. Before analyzing the data, melt curves were visually inspected for the presence of a single peak at melting temperature to ensure amplification specificity. Quantitative gene expression of each sample was normalized to glyceraldehyde-3-phosphate dehydrogenase (GAPDH) expression and quantified using the 2^−ΔΔCt^ method. Primer sequences used in the analysis are shown in Additional file 1: Table S1.

### Statistical analysis

Data are presented as the mean ± SD. The normality of data was checked using the Shapiro-Wilk normality test. Differential abundance analyses were conducted by one-way ANOVA with Tukey’s multiple comparisons test. All statistical analyses were conducted via Graph-Pad Prism 8 with statistical significance set at *P* < 0.05.

## Results

### Inhibitory effects of recombinant *B. bifidum *BGN4 strains on pro-inflammatory cytokines in vitro

**Fig. 2:**
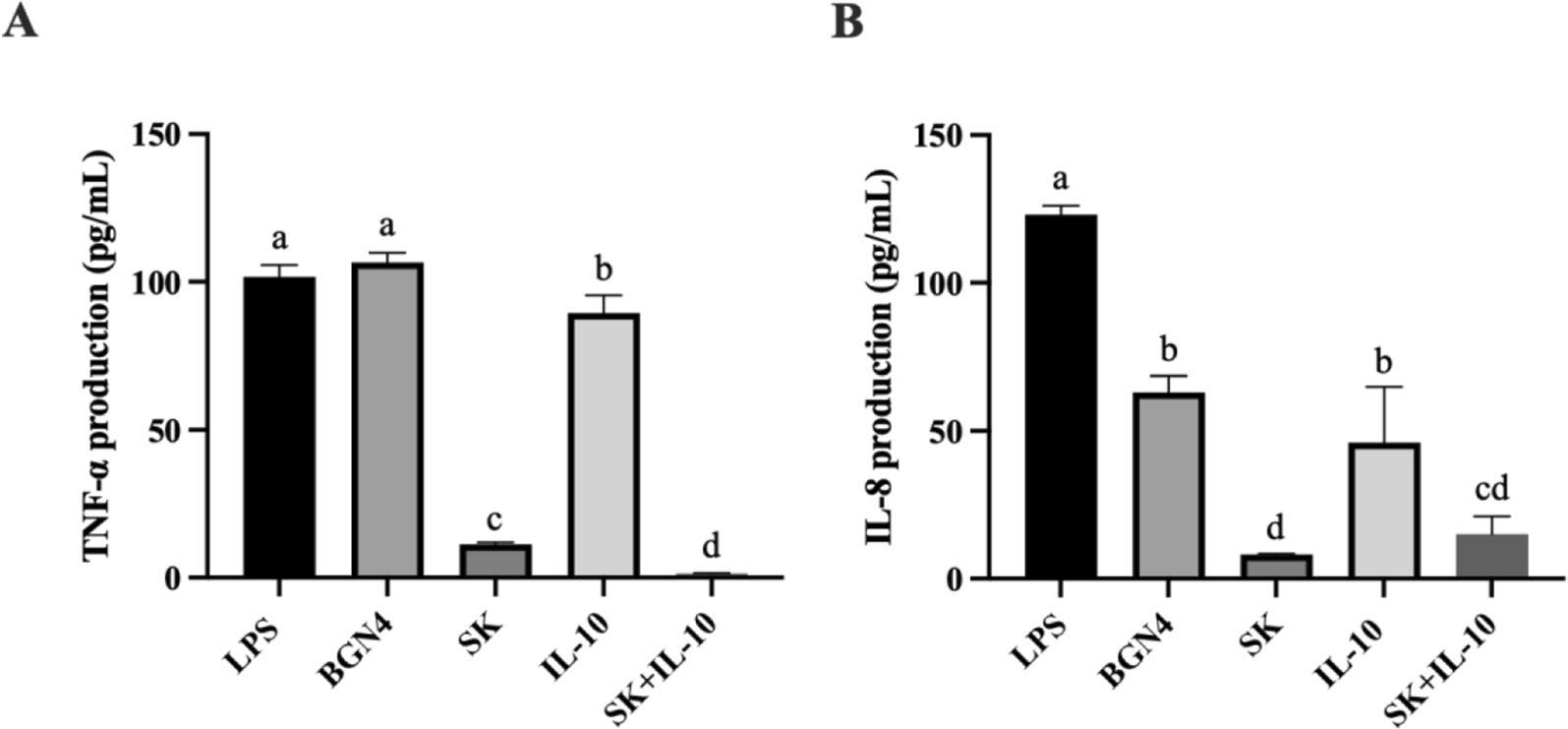
Production of TNF-α (**A**) and IL-8 (**B**) in lipopolysaccharide (LPS)-stimulated HT-29 cells when treated with cell-free supernatants (CFSs) of *B. bifidum* BGN4-SK, *B. bifidum* BGN4-pBESIL10 or their combination. Treatments with different letters are significantly different at *P* < 0.05. BGN4, *B. bifidum* BGN4; SK, *B. bifidum* BGN4-SK; IL-10, *B. bifidum* BGN4-pBESIL10

Compared with the LPS-treated control and the control strain *B. bifidum* BGN4 group, TNF-α production was significantly suppressed when cells were treated with a mixture of *B. bifidum* BGN4-SK and *B. bifidum* BGN4-pBESIL10 CFSs, followed by the *B. bifidum* BGN4-SK-only and *B. bifidum* BGN4-pBESIL10-only treatments (Fig. 2A). The combination of two recombinant *B. bifidum* strains resulted in synergistic inhibitory effects on the TNF-α production.

The generation of another pro-inflammatory cytokine IL-8 was significantly inhibited by *B. bifidum* BGN4-SK treatment, followed by the bacterial combination treatment. In terms of IL-8 suppression, the *B. bifidum* BGN4 control group also exerted anti-inflammatory effects but *B. bifidum* BGN4-pBESIL10 did not strengthen the efficacy and subsequently no synergistic effect between the recombinant bacteria was observed, as shown in Fig. 2B.

### Anti-inflammatory activities of recombinant *B. bifidum *BGN4 strains in a DSS-induced mouse colitis model

#### Clinical symptoms

**Fig. 3:**
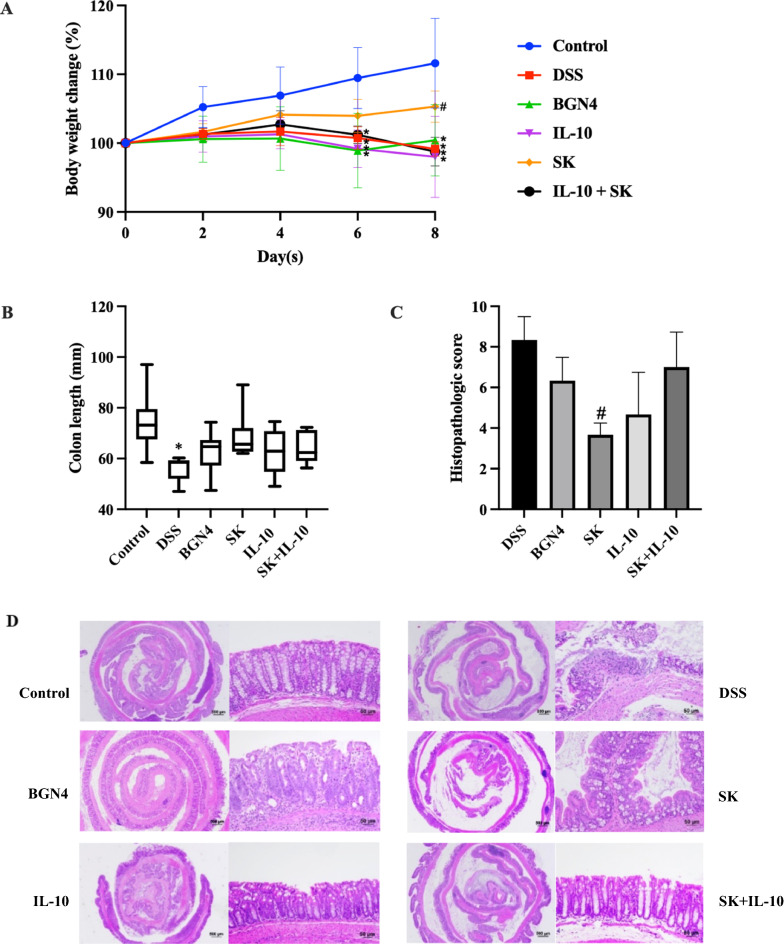
Effects of *B. bifidum* BGN4-SK, *B. bifidum* BGN4-pBESIL10 or their combination on clinical symptoms in DSS-induced colitis model. **A** Body weight change (%), **B** colon length (n = 8), **C** histopathologic score and **D** histological images of colonic tissues stained with H&E (n = 3). Data are expressed as mean ± SD. *Significant versus control. ^#^Significant versus DSS. *P < 0.05. The scale bars represent 500 μm (whole colon) and 50 μm (colonic segments). BGN4, *Bifidobacterium bifidum* BGN4; SK, *B. bifidum* BGN4-SK; IL-10, *B. bifidum* BGN4-pBESIL10

In this study, colitis inflammation was induced by 6-day oral administration of 2% DSS in drinking water. As shown in Fig. 3A, body weight slightly increased during the first four days in the DSS-received treatment group, and then significant body weight loss was observed in the four treatment groups (*B. bifidum* BGN4-SK group excepted), compared with control. After 2-day recovery period, the body weight in the *B. bifidum* BGN4-SK group was augmented and significantly larger than the DSS group. Colon length, as a critical indicator of inflammation severity, was significantly shortened in the DSS treatment compared to the control (Fig. 3B). To further assess ulceration and crypt loss in colon tissues, histological analysis was conducted and cumulative damage scores in the proximal, middle, and distal colon sections were recorded as histological scores, displayed in Fig. 3C. The histological scoring of colon pathology indicated that DSS-induced inflammation was significantly ameliorated in *B. bifidum* BGN4-SK-fed mice. According to the representative histological images of the colons presented in Fig. 3D, the control group showed intact crypt architecture and epithelial layers in the mucosa and submucosa, whereas loss of the entire crypt and epithelium disruption were observed with DSS treatment. Compared with the DSS group, the groups treated with the recombinant *B. bifidum* BGN4 strains showed less inflammation, indicating substantial protection of the colonic mucosa structure from DSS-induced damages. Of special note was the *B. bifidum* BGN4-SK-added treatment, which maintained crypt and goblet cell architectures and left intact mucosal and epithelial structures in the colonic tissues.

#### Antioxidant activities

**Fig. 4:**
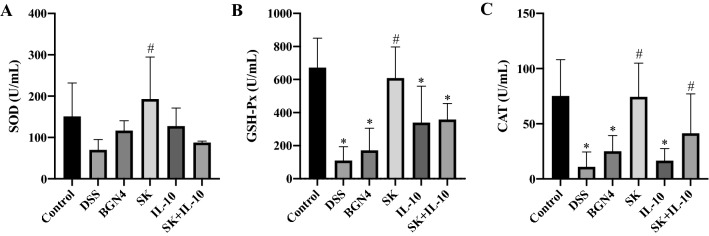
Superoxide dismutase (SOD) activity (**A**), glutathione peroxidase (GSH-Px) activity (**B**), and catalase (CAT) activity (**C**) in the blood serum of mice at the final day. Data are expressed as mean ± SD. *Significant versus control. ^#^Significant versus DSS. (n = 8). *P* < 0.05. BGN4, *Bifidobacterium bifidum* BGN4; SK, *B. bifidum* BGN4-SK; IL-10, *B. bifidum* BGN4-pBESIL10

SOD, GSH-Px and CAT are critical antioxidant enzymes against oxidative stress. As shown in Fig. 4, the DSS-only and *B. bifidum* BGN4/ BGN4-pBESIL10 administration treatments displayed significantly lower GSH-Px and CAT activities when compared to the control group, while SOD, GSH-Px and CAT activities in the *B. bifidum* BGN4-SK treatment were significantly higher than the DSS-only treatment. Additionally, the *B. bifidum* combination treatment displayed significantly higher CAT activity than the DSS group.

#### Intestinal barrier integrity

**Fig. 5:**
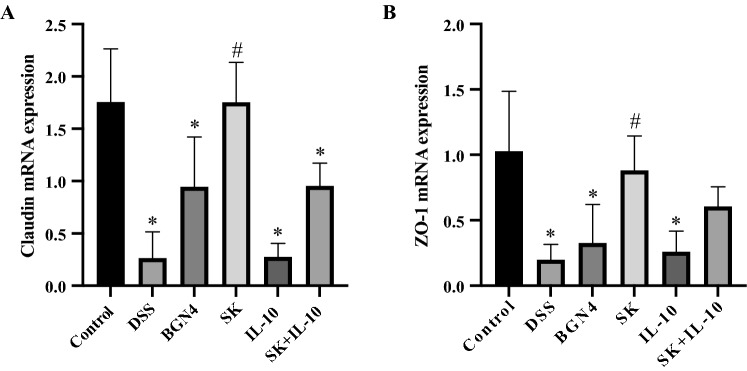
Relative mRNA expression of tight junction genes involving claudin (**A**) and ZO-1 (**B**). Data are expressed as mean ± SD. *Significant versus control. ^#^Significant versus DSS. *P* < 0.05. (n = 5). BGN4, *Bifidobacterium bifidum* BGN4; SK, *B. bifidum* BGN4-SK; IL-10, *B. bifidum* BGN4-pBESIL10

Relative mRNA expression of tight junction genes involving claudin and ZO-1 were examined to evaluate the integrity of the intestinal barrier in colon (Fig. 5). The expressions of claudin were significantly decreased in the four DSS-induced groups compared with the control group. Only the *B. bifidum* BGN4-SK treatment presented significantly higher claudin expression when compared to the DSS-only group. Similarly, *B. bifidum* BGN4-SK treatment showed significantly higher ZO-1 expression than the DSS treatment, while the expressions of ZO-1 significantly dropped in the DSS, *B. bifidum* BGN4 and *B. bifidum* BGN4-pBESIL10 groups, compared with the control.

#### MPO activity and cytokines in the colon

**Fig. 6:**
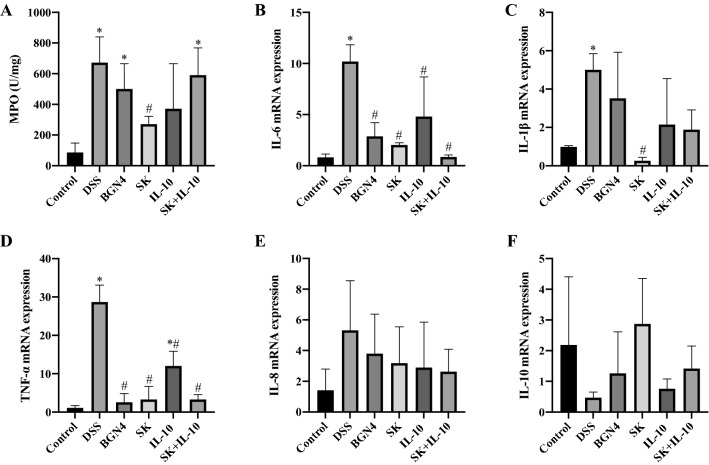
Myeloperoxidase (MPO) activities (**A**) and relative mRNA expression of pro-inflammatory cytokines (**B**–**F**). Data are expressed as mean ± SD. *Significant versus control. ^#^Significant versus DSS. (n = 5). *P* < 0.05. BGN4, *Bifidobacterium bifidum* BGN4; SK, *B. bifidum* BGN4-SK; IL-10, *B. bifidum* BGN4-pBESIL10

Colonic accumulation of MPO was measured as a neutrophil influx marker in the tissue. The activities of MPO in the DSS, *B. bifidum* BGN4 and recombinant bacteria combination groups were significantly higher than in the control group, and MPO in the *B. bifidum* BGN4-SK group was significantly decreased compared to the DSS group, as shown in Fig. 6A. The transcriptional levels of pro-inflammatory cytokines (IL-6, IL-1β, TNF-α and IL-8) and anti-inflammatory cytokine (IL-10) were investigated. The expression of IL-6 and TNF-α were inhibited in the four treatment groups compared with the DSS-only group, while the IL-1β expression was suppressed only in the *B. bifidum* BGN4-SK group when compared to the DSS-only treatment (Fig. 6B–D). Even though the inhibitory effect of the recombinant bacteria on IL-8 production was detected in vitro, it was not confirmed in this mouse model (Fig. 6E). In addition, the capability of anti-inflammatory IL-10 production in *B. bifidum* BGN4-pBESIL10 was not observed in vivo (Fig. 6 F).

## Discussion

In this study, the effects of two recombinant *B. bifidum* BGN4 strains and their combination were assessed on the development of inflammation and oxidative intestinal damage in vitro and in vivo. Both recombinant strain treatments led to anti-inflammatory effects in the cell line, and their combination appeared to have synergic effects. However, only *B. bifidum* BGN4-SK treatment significantly ameliorated colitis inflammation, boosted antioxidant enzymes, and protected the epithelial barrier in the DSS-treated mice.

Oral administration of DSS is toxic to colonic epithelial cells with increased apoptosis and decreased proliferation, leading to leakage of the epithelial barrier and mucosal invasion of intraluminal microorganisms [28]. Histological characteristics of colitis include epithelial distortions (i.e., crypt branching and shortening, reduction in crypt density and goblet cell numbers) and severe infiltration of inflammatory cells into intestinal walls [29]. Previous studies have indicated that probiotics are only slightly effective as IBD treatments (usually in less severe cases), because they generally act by reinforcing regulatory T cell responses in the mucosal immune system, which can be overwhelmed by an ongoing and robust inflammatory response [30]. Consistent with these previous studies, the group treated with the original *B. bifidum* BGN4 strain slightly alleviated DSS-induced inflammation, based on histological results.

Oxidative stress is seen as the main cause of tissue destruction in IBD patients, which emerges as a manifest imbalance between the production of ROS and their removal by antioxidants [13]. Previous studies have suggested the significance of antioxidant enzymes as a treatment approach for IBD [31–33]. Carroll et al. [16] reported that *Lactobacillus gasseri* secreting SOD alleviated inflammation in IL-10 knockout mice. Carmen et al. [34] revealed that recombinant *Streptococcus thermophilus* strains producing SOD/CAT enhanced anti-inflammatory activities both in vitro and in a mouse model of colitis. The benefits of these recombinant strains were mainly through their antioxidant mechanisms rather than an immunomodulating mechanism [35]. In addition, MPO is an enzyme in neutrophils, and its activity reflects the degree of neutrophil infiltration in intestinal inflammation [36]. More importantly, MPO catalyzes the formation of potent cytotoxic oxidants (i.e., hypochlorous acid), affecting the severity of inflammation [37]. In this study, we confirmed significant in vivo generation of antioxidant enzymes (SOD, GSH-Px and CAT) in the *B. bifidum* BGN4-SK treatment compared to the DSS group, which facilitated the attenuation of oxidative stress. Combined with reduced MPO activity and enhanced expression of tight junction genes, the *B. bifidum* BGN4-SK treatment maintained the functional and structural integrity of the colon tissues. Thus, the genetic modification of *B. bifidum* BGN4-SK to provide antioxidant production capability makes it superior to the wild-type strain in terms of colon tissue protection.

Cytokines are the critical signaling molecules of immune system [38]. TNF-α directly affects intestinal epithelial tissues and its secretion can lead to the disruption of the epithelial barrier and induction of apoptosis in epithelial cells [39]. IL-1ß and IL-6 are essential mediators of IBD progression [39]. IL-6 stimulates neutrophil chemotaxis, which is linked to necrosis in the colon [37]. Previous research reported that an antimurine IL-1ß antibody decreased IL-6 mRNA expression and alleviated pathological symptoms of DSS-induced colitis, suggesting that IL-1β targets itself and IL-6 for progressing colonic inflammation [40]. In this study, mRNA expressions of IL-6 and TNF-α were markedly reduced in the four bacterial treatments when compared to the DSS group, and only *B. bifidum* BGN4-SK treatment significantly decreased IL-1ß expression.

IL-10 is an essential anti-inflammatory cytokine, and IL-10-deficient mice have been reported to develop colitis spontaneously [41]. Steidler et al. demonstrated a 50% decrease in DSS-induced murine colitis using recombinant *Lactococcus lactis* secreting murine IL-10 [22], and then developed the first biocontainment system for *L. lactis* IL-10 strain. However, a phase IIA clinical trial did not reveal a statistically significant difference in mucosal healing with *L. lactis* IL-10 *versus* placebo [42]. Other previous studies implied that recombinant *L. lactis* and *B. bifidum* expressing IL-10 exerted mildly anti-inflammatory effects on DSS-induced colitis, but only for some clinical parameters [24, 42], which are consistent with our *in vivo* study. The low therapeutic efficacy of recombinant IL-10 indicates that a sustained and more mucosa-focused delivery is necessary for IL-10 to be effective against colitis [43]. Furthermore, using probiotics as vectors for IL-10 delivery to the mucosal surface ensures the release of IL-10 within the lumen without deep penetration into the tissues, which probably limits its anti-inflammatory effects [42]. IL-10 has broad immunoregulatory activities, including the inhibition of T_H_1 lymphocyte differentiation, promotion of regulatory T cell activities, and reduction of IL-12 release, which acts on immune cells in the lamina propria rather than at the mucosal surface [44, 45]. Hence, despite the very limited efficacy of *B. bifidum* BGN4-pBESIL10 observed in this study, it can potentially provide better results in immunologically driven chronic colitis models.

The biosafety of GMOs is a concern of the general public, and the biological issue is primarily the plasmid. The transfer of plasmids coding for antibiotic resistance has been reported both during food fermentation and *in vivo*, which indicates the potential for transmission of resistance genes to pathogenic species [46]. Lactobacilli and lactococci are increasingly recognized as reservoirs of antibiotic resistance genes [47], whereas *Bifidobacterium* spp. have low intrinsic and acquired resistance to antibiotics, suggesting they are safe live vectors for delivery of proteins in humans [48]. The genomic sequence of *B. bifidum* BGN4 published in GenBank (Accession No. CP001361.1) reveals this strain lacks plasmids capable of transferring antibiotic-resistance genes, the organism has been used as a food ingredient since 2000 [49, 50], and it is certified as Generally Recognized as Safe (GRAS) by U.S. Food and Drug Administration (FDA) (GRN No. 814).

In conclusion, this study demonstrated that both *B. bifidum* BGN4-SK and *B. bifidum* BGN4-pBESIL10 diminished the inflammatory levels in HT-29 cells. However, treatment by a recombinant strain expressing IL-10 presented very limited protective effects in the *in vivo* colitis model, while the *B. bifidum* BGN4-SK treatment effectively enhanced antioxidant capability, protected colonic epithelial integrity and inhibited colonic inflammation. In addition, there were no synergic effects between *B. bifidum* BGN4-SK and *B. bifidum* BGN4-pBESIL10 and the lower efficacy of the combination treatment was primarily due to the insufficient dose of the *B. bifidum* BGN4-SK strain. Hence, *B. bifidum* BGN4-SK is a potential candidate for IBD treatment. Clinical trials are a necessary next step to further confirm the potential for *B. bifidum* BGN4-SK treatment.

## Supplementary Information

**Additional file 1**: **Table S1. **Real-timepolymerase chain reaction (PCR) primer sequences.

## Data Availability

The datasets used and/or analysed during the current study are available from the corresponding author on reasonable request.
