# Supplementary material for: A recombinant Bifidobacterium bifidum BGN4 strain expressing the streptococcal superoxide dismutase gene ameliorates inflammatory bowel disease
Source: Microb Cell Fact. 2022 Jun 7;21:113. doi: 10.1186/s12934-022-01840-2 (PMC9172062; doi:10.1186/s12934-022-01840-2)
Supplement: Supplementary file 1 — Additional file 1: Table S1. Real-timepolymerase chain reaction (PCR) primer sequences. [file 12934_2022_1840_MOESM1_ESM.docx]

Additional file 1: Table S1. Real-time polymerase chain reaction (PCR) primer sequences

| **Gene** | **Forward primer (5’→3’)** | **Reverse primer (5’→3’)** | **Reference** |
| --- | --- | --- | --- |
| IL-6 | CTTCTTGGGACTGATGCTGGT | GGTCTGTTGGGAGTGGTATCC | (Yun et al., 2018) |
| IL-8 | TGGCTCTCTTGGCAGCCTTC | TGCACCCAGTTTTCCTTGGG | (Fang et al., 2010) |
| TNF-α | CATCTTCTCAAAATTCGAGTGACAA | TGGGAGTAGACAAGGTACAACCC | (Neel et al., 2013) |
| IL-1β | CCCAAGCAATACCCAAAGAA | GCTTGTGCTCTGCTTGTGAG | (Norimatsu et al., 2011) |
| ZO-1 | ATCCCTCAAGGAGCCATTC | CACTTGTTTTGCCAGGTTTTA | (Orlando et al., 2014) |
| Claudin-1 | GGCTTCTCTGGGATGGATCG | CCCCAGCAGGATGTAGCCCA | (Zhang et al., 2017) |
| GAPDH | AGGTCGGTGAACGGATTTG | TGTAGACCATGTAGTTGAGGTCA | (Xie et al., 2017) |

IL, interleukin; TNF-α, tumor necrosis factor-α; ZO-1, zonula occludens-1; and GAPDH, glyceraldehyde-3-phosphate dehydrogenase.
